# Confirmation of the Enhancement Effect of ANCE Treatment on Functional Recovery of Manual Dexterity From Primary Motor Cortex (M1) Lesion in Adult Macaque Monkeys

**DOI:** 10.1002/brb3.70986

**Published:** 2025-10-15

**Authors:** Camille Roux, Julie Savidan, Jean‐François Brunet, Jocelyne Bloch, Eric M. Rouiller, Eric Schmidlin, Mélanie Kaeser

**Affiliations:** ^1^ Department of Neurosciences, Section of Medicine, Faculty of Sciences and Medicine, Fribourg Center of Cognition University of Fribourg Fribourg Switzerland; ^2^ Cell Production Center (CPC) Lausanne University Hospital (CHUV) Lausanne Switzerland; ^3^ Department of Neurosurgery Lausanne University Hospital (CHUV) Lausanne Switzerland

**Keywords:** autologous cellular therapy, manual dexterity, motor cortex lesion, non‐human primate

## Abstract

**Introduction:**

A pilot study provided preliminary evidence indicating that peri‐lesional adult autologous neural cell ecosystem transplantation (so‐called ANCE) can enhance the functional recovery of manual dexterity in macaque monkeys following a unilateral lesion of the hand representation in the primary motor cortex (M1). As compared to five untreated control animals subjected to M1 lesions, the improvement of functional recovery was observed in our pilot study in two ANCE‐treated monkeys (also M1 lesioned). To strengthen these promising preliminary results, the present study aimed at introducing two additional ANCE monkeys, as well as one new control monkey, all subjected to M1 lesion.

**Methods:**

Manual dexterity was assessed based on the modified Brinkman board task, performed with the contralesional hand to retrieve small food pellets from slots using the precision grip (opposition of thumb and index finger).

**Results:**

As with the two pilot ANCE‐treated monkeys, the two new ANCE‐treated monkeys exhibited a rebound of functional recovery (a second plateau) associated to the treatment (after approximately 2–3 months delay post‐treatment), following a first plateau of spontaneous recovery. In contrast, all control monkeys (*n* = 6) exhibited a single plateau of functional recovery.

**Conclusion:**

The present study confirms that ANCE treatment significantly improves functional recovery from motor cortex lesions in adult macaques, with performance gains ranging from 20% to 40%. These findings support a possible future initiative to initiate clinical trials based on ANCE therapy.

## Graphical abstract

Together, an earlier pilot study and the current confirmatory study show that an autologous cellular therapy promotes beneficial, significant enhancement of manual dexterity recovery after lesion of the primary motor cortex.

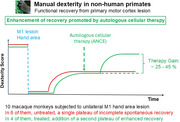

**Abbreviations**
ANCE: autologous neural cells ecosystem
CM: corticomotoneuronal
CS: corticospinal
GMP: good manufacturing practice
ICMS: intracortical microstimulation
M1: primary motor cortex
PM: premotor cortex
SMA: supplementary motor area

## 1 Introduction

Primates (humans and monkeys) exhibit a sophisticated motor ability to finely control finger movements, mostly under the control of the hand area of the primary motor cortex (M1 or F1). Manual dexterity depends on the direct corticospinal (CS) projection from layer V pyramidal neurons of M1 onto cervical motoneurons, which corresponds to the corticomotoneuronal (CM) projection system (Courtine et al. 2007), a prerogative of primates (e.g., Lawrence and Kyupers 1968; Luppino and Rizzolatti 2000; Dum and Strick 2002; Courtine et al. 2007; Lemon 2019, 2008; Bufacchi et al. 2023; but see also Kinoshita et al. 2012; Isa and Nishimura 2014; Yoshida and Isa 2018). The primary motor cortex (M1 or F1) has been subdivided into (i) an “old” M1, lacking CM cells and at the origin of CS axons connecting indirectly the spinal cord motoneurons through the intrinsic neuronal circuit of spinal interneurons, and (ii) a caudal region that has been designated as a “new” M1, containing the CM cells connecting directly the spinal cord motoneurons (Rathelot and Strick 2009). The major role played by M1 in manual dexterity is supported by the observation that, in intact monkeys, selective reversible pharmacological inactivation of the hand area in either the supplementary motor area (SMA) or premotor cortex (PM) does not substantially affect manipulation of small objects (Kermadi et al. 1997). Although the hand representations of SMA and PM also send CS projections (also some CM ones) to the cervical cord (He et al. 1993; He et al. 1995; Dum and Strick 1996; Rouiller et al. 1996; Borra et al. 2010; Morecraft et al. 2019), these projections are functionally less potent than those originating from M1 (Maier et al. 2002; Schmidlin et al. 2008; Boudrias et al. 2010b, 2010a). Nevertheless, the non‐primary motor hand representations in SMA or PM may become involved in case of unilateral M1 lesion, contributing to the mechanisms of spontaneous functional recovery of manual dexterity, though often incomplete (Liu and Rouiller 1999; Dancause et al. 2005, 2006; Mcneal et al. 2010; Hoogewoud et al. 2013; Morecraft et al. 2015; Orczykowski et al. 2018; Plautz et al. 2016, 2023; Moreau‐Debord et al. 2021).

Irrespective of the scenario underlying the functional recovery of manual dexterity following a unilateral lesion of the M1 hand area in adult macaque monkeys, the extent of spontaneous recovery remains largely incomplete (Liu and Rouiller 1999; Kaeser et al. 2011; Hoogewoud et al. 2013; Wyss et al. 2013; Savidan et al. 2017). As expected, the extent of the spontaneous functional recovery from the M1 lesion depends on the size of the M1 lesion; the smaller the lesion, the more extensive is the recovery of manual dexterity. There was preliminary evidence from pilot studies conducted in our laboratory showing that specific treatments such as anti‐Nogo‐A antibody treatment (*n* = 3 monkeys) or autologous cellular therapy (*n* = 2 monkeys) may enhance the functional recovery of manual dexterity after unilateral M1 lesion in adult macaque monkeys (Wyss et al. 2013; Kaeser et al. 2011, respectively).

**FIGURE 1:**
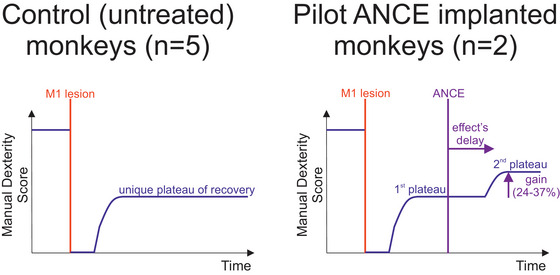
Reminder of the pilot data previously reported.^28^ In control monkeys (left panel; five monkeys), following unilateral M1 lesion and in the absence of ANCE treatment, the manual dexterity of the contralesional hand dropped to zero (left panel). After a few weeks, a spontaneous, incomplete functional recovery took place, reaching a unique plateau, reflecting the post‐lesion performance. In contrast (right panel; two monkeys), when the ANCE treatment was applied at a time point corresponding to the plateau of spontaneous recovery, with a delay of 2–3 months a rebound of functional recovery took place, reaching a second plateau of recovery. The latter yielded a gain of 24–37% of manual dexterity performance, as observed in these two pilot ANCE monkeys.

Regarding the autologous cellular therapy (referred to as *autologous neural cells ecosystem* (ANCE); see Brunet et al. 2003, 2005, 2009; Kaeser et al. 2011; Bloch et al. 2014, 2011; Borgognon et al. 2017, 2019; Fregosi et al. 2018, 2019), the evidence for enhanced functional recovery in the case of M1 lesion was limited due to the restricted number of two ANCE‐treated compared to five control (M1 lesion but no treatment) monkeys (Kaeser et al. 2011). The main evidence supporting an enhancement of functional recovery in the two ANCE‐treated monkeys is the occurrence of a second post‐lesion plateau of manual dexterity recovery, absent in the five control monkeys (Kaeser et al. 2011), as reminded in Figure 1. The goal of the present report was to confirm the preliminary evidence observed in the pilot data by extending the ANCE approach to two additional monkeys subjected to unilateral M1 lesions, as well as an additional control monkey. More specifically, does ANCE treatment promote the occurrence of the second plateau associated with the cellular transplantation in two additional monkeys? The present study meticulously followed the same experimental ANCE protocol as the previous one (Kaeser et al. 2011), while introducing the GMP (good manufacturing practice) requirements for the generation of the ANCE. Taking this into account, the present results represent a further step towards clinical trials in patients suffering from stroke or Parkinson's disease.

## 2 Material and Methods

### 2.1 Macaque Monkeys

**FIGURE 2:**
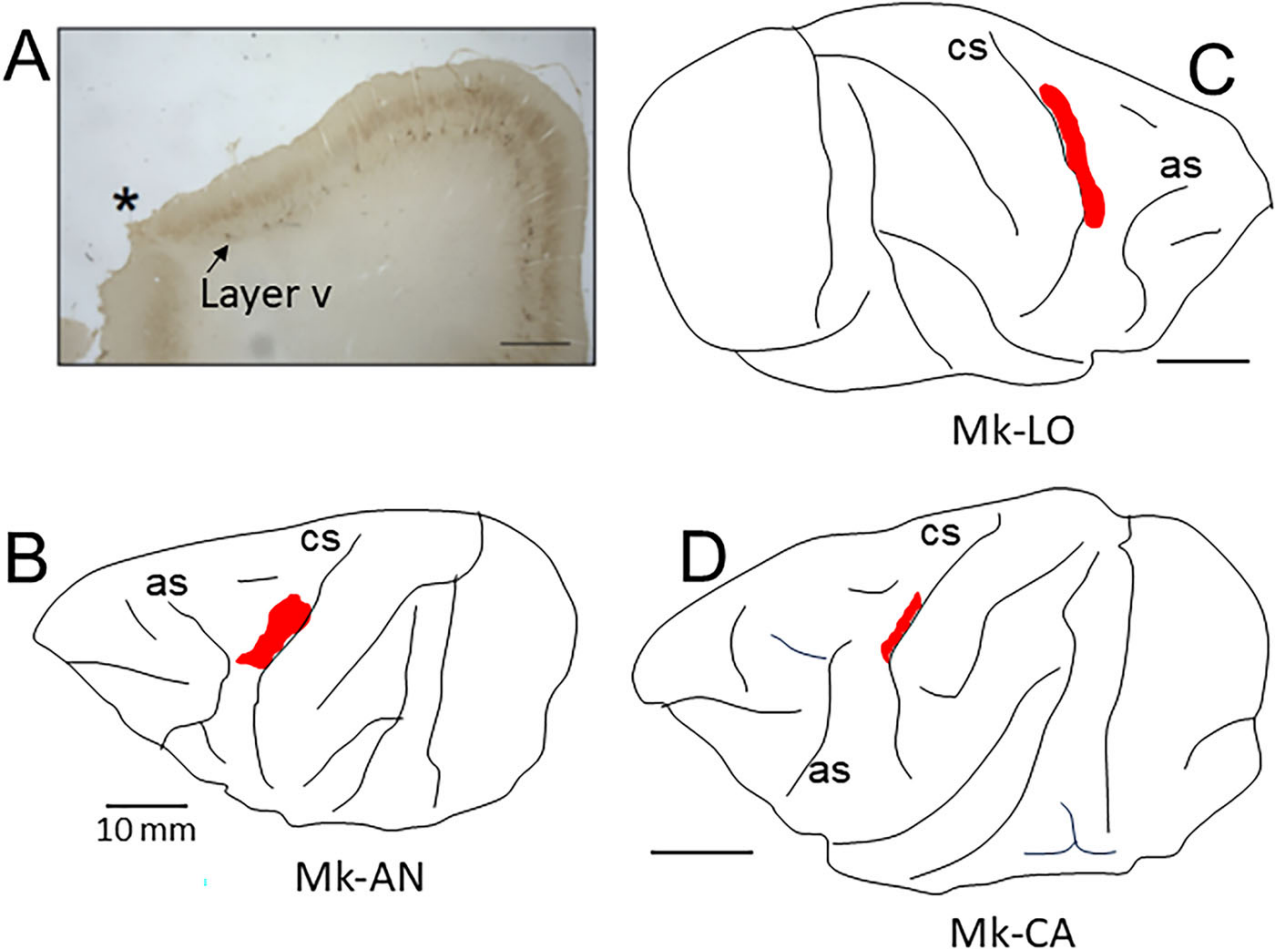
Representation of the cortical lesion targeted to the hand representation of M1 in the 3 newly introduced monkeys: Mk‐AN (panel B), Mk‐LO (panel C), and Mk‐CA (panel D), as projected on the surface of the corresponding hemisphere. The red area corresponds to the location and extent of the M1 lesion. Panel A is a photomicrograph taken from an SMI‐32 histological section in Mk‐LO intercepting the lesion in the middle of the rostro‐caudal extent of the lesion. The star indicates the lesioned zone on top of the rostral bank of the central sulcus, an area deprived of large pyramidal cells in layer V, destroyed as a result of the infusion of ibotenic acid. Starting from the arrow and in continuation to the right (medially towards the midline), the layer V clearly appears in the non‐lesioned territory characterized by the presence of large SMI‐32 positive pyramidal cells. Scale bars: 1 mm in panel A, 10 mm in panels B, C, and D. Abbreviations: as = arcuate sulcus, cs = central sulcus.

The present study involved a subsequent cohort of three adult macaque monkeys (*Macaca fascicularis*), added to the previously published report (Kaeser et al. 2011), itself based on seven monkeys. The individual data representative of these 3 newly introduced monkeys (Mk‐CA, Mk‐LO, and Mk‐AN) are listed in Table 1 (together with the data for the 7 previously published monkeys). Two new monkeys (Mk‐CA and Mk‐LO) were subjected to the ANCE treatment after the M1 lesion, whereas the third new monkey (Mk‐AN) was an additional control animal (M1 lesion but no treatment). The unilateral M1 lesion, mostly restricted to the hand representation, was induced by intracortical infusion of ibotenic acid, as previously described (Liu and Rouiller 1999; Kaeser et al. 2011, 2010; Wyss et al. 2013; Savidan et al. 2017; see below for more details). The manual dexterity performances of these three new monkeys in early phases when they were still intact are reported in a previous report (Kaeser et al. 2014). The experimental and detention conditions with respect to compliance with ethical and legal guidelines were described in detail in several previous publications from this laboratory (Kaeser et al. 2011, 2010, 2014, 2013; Wyss et al. 2013; Savidan et al. 2017; see also www.unifr.ch/spccr/about/housing). The unilateral lesion in Mk‐CA was restricted to the M1 hand area to specifically affect the motor control of the digits of the opposite hand. The lesion was preceded by M1 mapping using intracortical microstimulation (ICMS) to identify the sites of ibotenic acid infusions. Described in detail previously (Savidan et al. 2017), the procedure used a chronic head chamber implanted on the skull, above M1. The chamber contained two Tecapeek grids, each comprising an 8 by 8 hole configuration, with a separation of 1 mm between adjacent sites. These holes allowed perpendicular insertions of microelectrodes used to elicit ICMS at various depths in M1. An extensive electrophysiological mapping of M1 guided the selection of specific ICMS sites where movements of the digits were elicited at low threshold in the contralateral hand for subsequent infusions of ibotenic acid, producing a permanent lesion of M1 (Liu and Rouiller 1999; Kaeser et al. 2011; Wyss et al. 2013; Savidan et al. 2017). A comparable ICMS mapping of M1, preceding the lesion with ibotenic acid, was also conducted in Mk‐JU, Mk‐GE, Mk‐RO, Mk‐CE, Mk‐BI, Mk‐JO, and Mk‐JA. Overall, eight out of the ten monkeys (Table 1) underwent an M1 electrophysiological mapping before lesion. In only two monkeys (Mk‐AN and Mk‐LO), the M1 lesion was guided by anatomical landmarks, mainly the typical trajectory of the central sulcus ventral to the hand area (Figure 2). The excitotoxic ibotenic acid injections were performed using a 10 µL Hamilton micro‐syringe, whose tip was positioned at the selected ICMS sites. Table 1 lists for each monkey the number of ibotenic acid infusion sites, as well as the total volume injected. The methods to delineate the lesion extent on histological sections stained for the neurofilament SMI‐32 and the calculation of the lesion volume were reported earlier (Wyss et al. 2013; Contestabile et al. 2018). Briefly, frontal sections of the monkey's brain were analyzed and reconstructed using Neurolucida 9 (MBF Bioscience), with a computer‐interfaced Olympus BX40 microscope, a computer‐controlled motorized stage (Märzhäuser Wetzlar GmbH, type EK 3275×50), and a digital camera (Olympus U‐PMTVC). On each relevant section, the lesioned cortical zone in M1 was defined as the territory characterized by a loss of SMI‐32 positive neurons in layer V (Figure 2A), perpendicularly extended up to the surface and down to the white matter limit. The total M1 lesion volume was then derived by integrating the lesioned zones on the consecutive individual frontal sections based on the Cavalieri's method (Pizzimenti et al. 2007). The M1 lesion volumes are listed for each monkey in Table 1.

**TABLE 1:** Overall individual monkeys’ data (current study and monkeys from Kaeser et al. 2011).

	Control Group—M1 lesion—Untreated						ANCE treated Group—M1 lesion			
	Mk‐JU^1^	Mk‐GE^1^	Mk‐RO^1^	Mk‐CE^1^	Mk‐BI^1^	Mk‐AN	Mk‐JO^2^	Mk‐JA^2^	Mk‐CA	Mk‐LO
Species	M.fasc.	M.fasc.	M.fasc.	M.fasc.	M.fasc.	M.fasc.	M.fasc.	M.fasc.	M.fasc.	M.fasc.
Sex	Male	Female	Male	Male	Male	Female	Male	Male	Female	Female
Age sacrifice (rounded 0.5 y)	6	6	4.5	5.5	6	14.5	4	5	12	13
Age at lesion (rounded 0.5 y)	5	5	4	4.5	5	14	3.5	4	11	11.5
Weight (at lesion day)	3.6	2.8	3.2	3.8	5	6.4	3.4	4.1	5.5	4
Treatment	None	None	None	None	None	None	ANCE	ANCE	ANCE	ANCE
Number of ANCE sites	—	—	—	—	—	—	1	10	8	4
Estimated number. of re‐implanted cells	—	—	—	—	—	—	250 k	750 k	2’820 k	2’610 k
Interval lesion—ANCE impl.	—	—	—	—	—	—	15 days	127 days	130 days	220 days
Lesioned hemisphere	Right	Left	Left	Left	Left	Left	Left	Left	Left	Right
Type of lesion	Ibo. acid	Ibo. acid	Ibo. acid	Ibo. acid	Ibo. acid	Ibo. acid	Ibo. acid	Ibo. acid	Ibo. acid	Ibo. acid
Total volume of lesion (mm^3^) in the gray matter	63	48.7	14	112.8	20.1	27.7	30	20.5	22	19.1
Number of ibo. acid sites	21	13	12	21	29	12	10	38	14	18
Volume ibo. acid (µL)	40	13	18	40	29.7	24	15	38	18.1	29
Volume lesion in post central gyrus (mm^3^)	0	7.6	0	10.1	0	0	0	0	0	0
Number of functional recovery plateau^a^	1	1	1	1	1	1	2	2	2	2
Delay of ANCE effect^a^	—	—	—	—	—	—	65 days	96 days	105 days	88 days
% FR tot score first plateau^a^	39%	38%	98%	42%	74%	65%	35%	61%	50%	65%
% FR ver score first plateau^a^	46%	57%	100 %	59 %	94 %	70 %	55 %	67%	76%	64%
% FR hor score first plateau^a^	29%	11%	90%	9%	36%	55%	0%	54%	25%	67%
% FR tot score second plateau^a^	—	—	—	—	—	—	59%	98%	67%	92%
% FR ver score second plateau^a^	—	—	—	—	—	—	83%	93%	71%	93%
% FR hor score second plateau^a^	—	—	—	—	—	—	25%	100%	61%	92%
**% gain first‐second plateau Hor**	—	—	—	—	—	—	**25%**	**46%**	**36%**	**25%**

All monkeys were subjected to a motor cortex injury (M1 in the hand representation, unilaterally).

Abbreviations: ANCE = autologous neural cell ecosystems (autologous adult neural progenitor cells re‐implanted in the intact peri‐lesion territory in M1); FR = functional recovery, Hor = horizontal slots, Ibo. acid = ibotenic acid (with indication of the number of sites where it was injected in M1); Tot = total; Ver = vertical slots.

^1^Derived from previous behavioral studies: Liu and Rouiller 1999; Kaeser et al. 2010; Bashir et al. 2012; Hamadjida et al. 2012; Hoogewoud et al. 2013; Wyss et al. 2013.

^2^Derived from a previous behavioral ANCE study: Kaeser et al. 2011.

^a^ Behavioral data derived from the Modified Brinkman board task.

The experiments conducted on the monkeys Mk‐CA, Mk‐LO, and Mk‐AN, newly introduced in the present report, were covered by the veterinary authorizations No. 192‐07E, 206/08, and 2014‐FR‐42E, delivered by the cantonal (Fribourg) and federal (Swiss) veterinary authorities. For the other seven monkeys considered as well in the present study and listed in Table 1, the relevant veterinary authorizations were mentioned in previous publications from this laboratory (Kaeser et al. 2011; Wyss et al. 2013).

### 2.2 Manual Dexterity Behavioral Task

In the present study, manual dexterity was quantified in the three monkeys Mk‐CA, MK‐LO, and Mk‐AN using the “modified Brinkman board” task, as done earlier in the seven other monkeys and as described in detail previously (Rouiller et al. 1998; Liu and Rouiller 1999; Freund et al. 2006, 2009; Kaeser et al. 2011, 2010, 2014, 2013; Schmidlin et al. 2011; Chatagny et al. 2013; Wyss et al. 2013; Badoud et al. 2017; Borgognon et al. 2019). Video sequences attached to the present report also illustrate the “modified Brinkman board” task.

**FIGURE 3:**
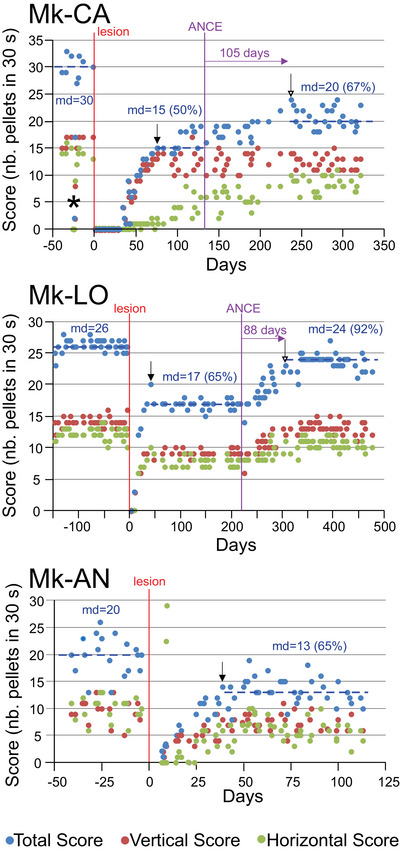
Modified Brinkman board data for the newly introduced three monkeys in the current study (Mk‐CA, Mk‐LO, and Mk‐AN). The score (number of pellets successfully retrieved in 30 s), indicative of the manual dexterity, was plotted as a function of time (daily behavioral sessions) for the contralesional hand. All three monkeys were subjected to a unilateral lesion of the hand area in M1 (red vertical line corresponding to day zero in the abscissa; see also Figure 1). Mk‐CA and Mk‐LO received the ANCE treatment at the time point indicated by the vertical purple line. The vertical arrows indicate the day of onset of the first and second plateaus, based on the criteria reported earlier (Kaeser et al. 2011). The scores are given for the vertical slots of the Brinkman board (red circles) and the horizontal slots (green circles). The blue circles are for the total scores (sum of the vertical and horizontal scores). The median score values (md) are indicated for the pre‐lesion phase, as well as during the first and second plateaus. The percentages indicate the extent of functional recovery for the first and second plateaus.

**FIGURE 4:**
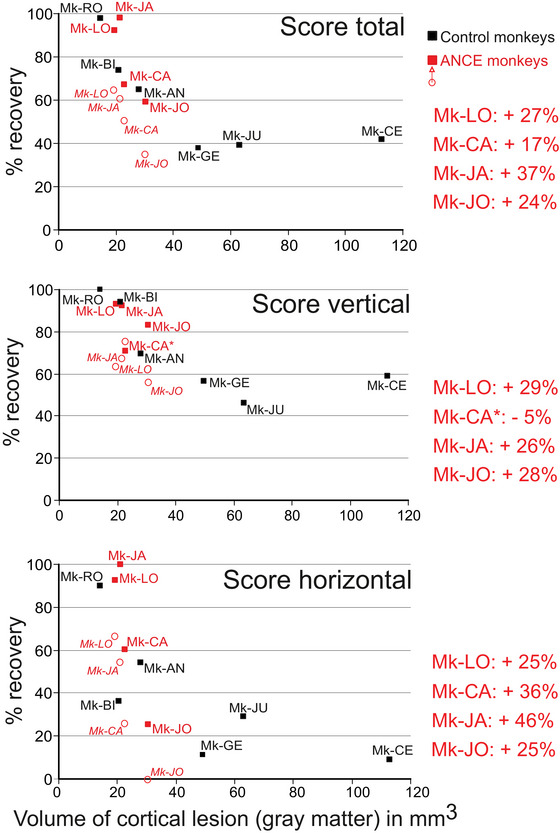
Summary of the behavioral data derived from the modified Brinkman board task for the three newly introduced monkeys in the current study, as well as the seven monkeys published earlier (Kaeser et al. 2011). The total number of monkeys is thus ten. The three plots from top to bottom show the functional recovery in % for the total score (top), vertical score (middle), and horizontal score (bottom) as a function of the lesion volume (in mm^3^). The control monkeys are in black, while the ANCE monkeys are in red. The six control monkeys are depicted each by a single black square, corresponding to their unique plateau of spontaneous functional recovery in thw absence of treatment. The four ANCE monkeys are each represented by two red symbols, an open circle for the functional recovery at the first plateau (spontaneous recovery) and a red solid square for the functional recovery at the second plateau (ANCE‐enhanced functional recovery). The identity of each monkey is indicated next to each symbol (except for Mk‐CA in the middle panel, as indicated by the asterisk with one ID next to the two symbols corresponding to this animal). On the right of each plot, in percent, the gain in functional recovery provided by the treatment for each ANCE monkey.

The modified Brinkman board task tests the ability of the monkeys to grasp small food pellets from spatially restricted wells with each hand separately, requiring them to perform the precision grip defined as an opposition of the thumb and index finger. The Brinkman board comprises 25 vertically and 25 horizontally orientated wells, corresponding to a total of 50 wells. The precision grip can be executed in the vertical wells, keeping the hand in its neutral posture, whereas horizontal slots require a wrist/arm rotation (ulnar or radial deviation) in addition to the precision grip (Kaeser et al. 2013). Consequently, the horizontal wells may be considered as more challenging than the vertical wells. The manual performance was expressed as a score given by the number of pellets retrieved during the first 30 s of the task. The score was computed separately for the vertical and horizontal wells, while the total score was the sum of the two. In order to quantify the functional recovery post‐lesion, after reaching a plateau of recovered performance, the median post‐lesion score was divided by the median pre‐lesion score, multiplied by 100, yielding a percentage of functional recovery of manual dexterity (see Figures 3 and 4).

### 2.3 ANCE Production and Re‐implantation

The production of the ANCE was the same as previously reported in detail (Kaeser et al. 2011; Brunet et al. 2003, 2005, 2009). In particular, the ANCE was derived from a biopsy of cortical tissue at the level of the prefrontal cortex in the same monkey, performed several months before the M1 lesion, as previously reported (Kaeser et al. 2011; Badoud et al. 2017). However, the protocol was implemented in the present study under good manufacturing practices (GMP). Instead of proceeding under a classical laminar flow hood and growing cells in a conventional incubator, the productions of ANCE were done into a bio‐confinement cabinet called Isocell pro 1.8 (Euroclone, Italy). This specific equipment includes the working area for manipulation, the incubator with rotating agitation for cell growing, the microscope for cell observation, and the automatic batch reporting. These GMP conditions did not influence cell growth or ANCE formation.

After completion of the cell cultures, the ANCE were reimplanted in the same monkey from which the biopsy was obtained (autologous transplantation), at a time point corresponding to a well‐established first plateau, reflecting the spontaneous functional recovery level (Figures 1 and 3), 130 days and 220 days post‐lesion in Mk‐CA and Mk‐LO, respectively. As previously reported (Kaeser et al. 2011), the ANCE were re‐implanted at multiple sites in M1 in the vicinity of the lesioned territory (see Table 1), in intact gray matter territories.

## 3 Results

### 3.1 Unilateral Lesion in M1 Hand Representation

The characterization of the hand representation in M1, as well as the lesion properties, was illustrated for the seven monkeys previously reported in earlier reports from this laboratory (Kaeser et al. 2010; 2011; Wyss et al. 2013; Fregosi et al. 2018). The volume of the M1 lesion in each animal was determined histologically (Table 1). Three monkeys (Mk‐CE, Mk‐JU, and Mk‐GE) exhibit an M1 lesion clearly larger than the others. These three monkeys were the first ones to be lesioned, 2–3 decades ago (Liu and Rouiller 1999; Wyss et al. 2013), representing pilot animals. In subsequent experiments, it was attempted to perform more precise and more restricted lesions, focused on the M1 hand area. A representation of the location and extent of the M1 lesion is provided only for the three newly introduced monkeys in the present confirmation study (Figure 2). The M1 lesion in Mk‐AN and Mk‐LO was mostly located in the so‐called “old M1” area, whereas in Mk‐CA the lesion affected mostly the “new M1” area, along the rostral bank of the central sulcus, referring to the nomenclature introduced by Ratelot and Strick (2009).

### 3.2 Modified Brinkman Board Task

#### 3.2.1 ANCE Enhanced Functional Recovery

Previous pilot data have shown that, after unilateral M1 lesion, control (untreated; n = 5) monkeys subjected to M1 lesion exhibited a spontaneous, incomplete functional recovery of manual dexterity for the contralateral affected hand in the form of a unique post‐lesion plateau reached after a few weeks/months (Figure 1 left panel; Table 1), remaining at a stable level, at least for a year or more (Kaeser et al. 2011). In contrast, M1‐lesioned monkey reimplanted with ANCE exhibited a rebound of recovery, reflected by a second additional plateau (Figure 1, right panel; Table 1). Such enhancement of functional recovery occurred with a delay of about 2–3 months post ANCE reimplantation (Kaeser et al. 2011), although it was observed in only two animals (Mk‐JO and Mk‐JA; see Table 1). Moreover, the ANCE re‐implantation was performed at variable time points post‐lesion and in one case was performed in two steps. The goal of the present study was to replicate these pilot data (second plateau of recovery) on two additional ANCE monkeys (Mk‐CA and Mk‐LO), following a more standardized protocol.

In a third additional monkey (Mk‐AN), corresponding to a control animal (M1 lesion, untreated), the behavioral data reproduced the same pattern of spontaneous functional recovery as observed previously in the 5 control animals from the pilot study, namely the occurrence of a unique plateau post‐lesion (Figure 3). Mk‐AN exhibited a fairly large performance variability among the daily sessions, with, however, a unique plateau of functional recovery corresponding to 65% with respect to pre‐lesion performance (Figure 3). In contrast, the two new animals subjected to the M1 lesion and recipients of the ANCE treatment (Mk‐CA and Mk‐LO) exhibited a second plateau of functional recovery post‐lesion (Figure 3). These data confirm the findings from the two pilot monkeys previously reported (Kaeser et al. 2011). In more detail, and based on the total score, the spontaneous functional recovery of manual dexterity, given by the first post‐lesion plateau, represented 50% and 65% of the pre‐lesion total score in Mk‐CA and Mk‐LO, respectively. Their second post‐lesion plateau for the total score reached 67% and 92% (with respect to pre‐lesion score), corresponding to a rebound of recovery of 17% and 27% in Mk‐CA and Mk‐LO, respectively (see also Figure 4). Considering the vertical score, the gain in recovery provided by the second plateau was none in Mk‐CA (actually a slight decrease of 5%), whereas it was 29% in Mk‐LO (see also Figure 4). For the horizontal score, the rebound of the second plateau was 36% in Mk‐CA and 25% in Mk‐LO (see also Figure 4).

Video sequences 1 and 2 illustrate the manual dexterity performance of Mk‐CA and Mk‐LO, comparing the pre‐lesion phase, the acute post‐lesion phase (a few days after the M1 lesion), the spontaneous recovery phase (first plateau), and the second plateau phase with the rebound of functional recovery related to the ANCE treatment. These videos highlight qualitatively the enhancement of manual dexterity offered by a 17% to 27% additional improvement (based on the total score in Mk‐CA and Mk‐LO, respectively). A more detailed analysis of these video sequences further illustrates the gain of performance at the second plateau as compared to the first plateau. The monkey Mk‐CA retrieved the 50 pellets at the second plateau, while, at the same time point (after 1 min and 47 s) of the first plateau, there were still 22 pellets left on the board. This corresponds to a gain of 44% considering the number of slots emptied in the time window necessary to fully empty the board at the second plateau. In monkey Mk‐LO, the board was terminated at the second plateau (50 slots emptied after 1 min and 50 s), while, at the same time point, there were still 13 pellets left on the board at first plateau: a gain of 26% in that case. These data, based on the full completeness of the task (50 pellets retrieved in total instead of the score in the first 30 s as in Figure 3), may introduce a bias due to a loss of motivation of the monkey to retrieve pellets in the subsequent phase of the test (after 1 min or so).

**VIDEO 1:** The video shows the pellet retrieval performance of the contralesional hand (unilateral M1 lesion in the hand area) observed for the modified Brinkman board task in monkey Mk‐CA in representative daily sessions. The performance is illustrated at four time points: (i) Pre‐lesion; (ii) Post‐lesion in the acute phase (2 days after the M1 lesion); (iii) Post‐lesion at plateau 1 of functional recovery (3 months after the M1 lesion); and (iv) Post‐lesion at plateau 2 of functional recovery (1 year after the M1 lesion). Note the complete loss of manual dexterity (precision grip) immediately after the lesion. The comparison between plateau 1 and 2 shows qualitatively the gain of functional recovery (+ 17% of score; see Figure 3) associated with the ANCE treatment. Note that the monkey did not fully recover at plateau 2, as illustrated by a few errors, especially in the horizontal slots (pellets dropped from the board). Note also that in the acute phase post‐lesion, the monkey tried a few times to cheat by using the ipsilesional (left) hand (prevented from doing so by the experimenter). Video available at: https://tube.switch.ch/videos/wpZRKFFPCQ.

**VIDEO 2:** Same as Video 1, but for monkey Mk‐LO. The four time points are as follows: (i) Pre‐lesion; (ii) Post‐lesion in the acute phase (1 week after the M1 lesion); (iii) Post‐lesion of plateau 1 of functional recovery (1 month after the M1 lesion); and (iv) Post‐lesion at plateau 2 of functional recovery (1 year after the M1 lesion). Note that in the acute phase post‐lesion, the monkey tried once to cheat by using the ipsilesional (right) hand. Video available at: https://tube.switch.ch/videos/wsLXhuAnaC.

#### 3.2.2 Temporal Motor Strategy and ANCE Effect Delay

The two videos illustrate a change of temporal strategy in the form of the spatial sequence to visit the different zones of the board. For instance, at the first plateau, Mk‐LO visited the first slots in the center of the board and then moved to the left part of the board before terminating at the right part of the board; at the second plateau, the sequence was rather a progressive shift from the top area to the bottom area of the board. In Mk‐CA, the sequence to visit the board went from center to right and then to the left part of the board at the second plateau; at the first plateau, the sequence was largely random.

The delay of occurrence of the second plateau in Mk‐CA and Mk‐LO was 105 days and 88 days, respectively (Figure 3), possibly representing the time for the ANCE transplant to exert its action. These delays are largely in line with those observed in the two pilot monkeys (ranging from 65 days to 96 days: Kaeser et al. 2011), although the re‐implantation protocols were not identical (see discussion below).

#### 3.2.3 Types of Movements to Retrieve Pellets

By comparing the video sequences pre‐ and post‐lesion, we aimed to provide more qualitative insights into the recovery process. This analysis allowed us to determine whether the movements to retrieve pellets post‐lesion at the plateau were similar to the original movements pre‐lesion or if the recovery involved some compensatory strategies. All monkeys at the pre‐lesion plateau of performance used a typical precision grip sequence, grasping the pellets between the tips of the index finger and the thumb. Usually, the index finger entered the slots before the thumb. In a few monkeys, the thumb entered first for the horizontal slots and only in about 10–20% of trials. Post‐lesion, at plateaus 1 and 2, the recovered sequence of movements was largely comparable to the original pre‐lesion ones. The recovered precision grip, however, exhibited occasionally a diminished degree of independence of the index finger with respect to digits 3–5, resulting in a slower performance overall (in line with the incomplete functional recovery). In the ANCE‐treated monkeys, the loss of independence was decreased at plateau 2 as compared to plateau 1.

There were, moreover, minor but noticeable differences in a few animals. As previously reported (Hoogewoud et al. 2013), there were sometimes slight changes in the preferred wrist angle when approaching horizontal slots, comparing pre‐lesion and post‐lesion trials. These angle adaptations were more present in the monkeys with limited functional recovery, as compared to monkeys with better recovery. In Mk‐CE, for instance, the balance between wrist abductions versus wrist adductions was slightly modified post‐lesion. Whereas the abductions were more frequent, we have observed an increase of wrist adductions post‐lesion (35% vs. 25% pre‐lesion). In Mk‐JO, there was a dominance of wrist adductions pre‐lesion (80%), whereas post‐lesion wrist abductions and adductions were equally frequent.

In Mk‐AN, for the horizontal slots, the index finger or the thumb of the right hand was first inserted depending on the position of the corresponding slot on the board. At the pre‐lesion period, the thumb was inserted first in the right half of the board and the index finger first for the left half of the board. In contrast, at the post‐lesion plateau, the thumb‐first insertions increased while the index‐first insertions decreased. The situation was reversed in Mk‐CE as compared to Mk‐AN, with a nearly total loss of thumb‐first insertions post‐lesion, whereas it represented about 15% of first insertions pre‐lesion (for 85% index‐first insertions). In contrast to the other animals, the monkey with the largest M1 lesion and a fairly poor functional recovery (Mk‐CE), especially for horizontal slots, exhibited a clear change of grasp pattern to retrieve the pellets from both slots’ orientations. Instead of opposing the tips of the thumb and index finger as observed pre‐lesion, Mk‐CE rather performed an opposition of the index tip with the proximal part of the thumb and/or the hand palm at the plateau post‐lesion.

### 3.3 Data Summary

The manual dexterity data for all ten monkeys are summarized in Figure 4 (see also Table 1). This figure illustrates the percentage of functional recovery of manual dexterity in relation to the volume of M1 lesion in the contralateral hemisphere. The data are represented in three different plots for the total, vertical, and horizontal scores (top to bottom, respectively). The seven control monkeys (M1 lesion, no treatment, and depicted with a single black square), exhibiting a unique plateau of functional recovery, showed progressive decline of functional recovery for increasing lesion volumes, as expected. The four ANCE monkeys are represented in red by two symbols each, with an open circle for the functional recovery at the first plateau, while the red square is for the functional recovery at the second plateau. The percentages on the right part of Figure 4 indicate the gain in percentage of functional recovery from the first to the second plateau. The horizontal score, reflecting the most challenging movement sequence, exhibited a specific improvement from the first to the second plateau ranging from 25% to 46% across the four ANCE monkeys.

## 4 Discussion

### 4.1 Contribution of the Present Study

The present study provides further evidence for a beneficial effect of the ANCE treatment to enhance the functional recovery of manual dexterity from unilateral M1 lesions in adult macaque monkeys. The two newly introduced ANCE‐treated subjects (Mk‐CA and Mk‐LO) fully corroborate the preliminary findings reported previously on the monkeys Mk‐JO and Mk‐JA (Kaeser et al. 2011). Overall, the two studies (Kaeser et al. 2011 and the current one) are based on a relatively large cohort of ten monkeys, considering the ethical limitations to conducting experiments on non‐human primates, especially those involving lesions of the central nervous system. From these ten monkeys, all subjected to unilateral lesion of the M1 hand representation, two groups were formed: (i) a control group of six monkeys without treatment, reflecting the extent and features of spontaneous functional recovery; and (ii) a treated group of four monkeys, re‐implanted with the ANCE. The first strong evidence for a beneficial effect of the ANCE treatment lies in the dramatic difference of the pattern of functional recovery between the two groups. In the control group (*n* = 6), all monkeys exhibited a single post‐lesion plateau of incomplete functional recovery, without the occurrence of a rebound (second plateau). In sharp contrast, all monkeys (*n* = 4) of the ANCE‐treated group systematically exhibited a second plateau, corresponding to a rebound of functional recovery. Such a strict dissociation between the two groups of monkeys with regard to the occurrence of a second plateau provides solid quantitative evidence supporting the beneficial role played by the ANCE therapy. A second line of strong evidence relates to the temporal dynamics of the ANCE effect. In the four ANCE‐treated monkeys, the delay between the re‐implantation and the onset of the second plateau was largely consistent across the monkeys Mk‐CA and Mk‐LO (current study) as well as Mk‐JO and Mk‐JA (Kaeser et al. 2011), ranging from 65 days to 105 days. In more detail, in the three monkeys (Mk‐CA, Mk‐LO, and Mk‐JA) in which the reimplantation took place when the first plateau was established, the delay was 105 days, 88 days, and 96 days, respectively. Considering some biological variation, it can be concluded that these three monkeys exhibited very comparable time courses of action for the ANCE. The fourth monkey (Mk‐JO) exhibited a shorter delay of 65 days (Kaeser et al. 2011), which may be explained by the time course of the re‐implantation, which took place very early during the acute phase of total paralysis, before the onset of any recovery, about one month before the first plateau (Kaeser et al. 2011). This different schedule may explain the shorter delay observed in Mk‐JO. Another difference concerning the first monkey tested for ANCE therapy in the series, Mk‐JO, is that it received the transplanted cells at a single site (Table 1) and in a smaller quantity than the other three ANCE treated monkeys. This may explain the smaller degree of functional recovery of manual dexterity observed for Mk‐JO as compared to the other three ANCE‐treated monkeys. Interestingly, the shorter delay in Mk‐JO mentioned above is not associated with a better rebound of functional recovery, as compared to the other three monkeys. This provides a highly encouraging outcome for future clinical applications, indicating that a re‐implantation occurring later in time (even after 4 to 7 months post‐lesion; see Figure 3) remained as efficient as an earlier re‐implantation.

An important issue is the extent of the benefit of functional recovery offered by the ANCE treatment, evidenced by the second plateau of recovery (as observed in the modified Brinkman board task). In other words, how efficient is the ANCE treatment? The answer is an improvement in manual dexterity in the order of 20% to 40%, though with some individual variations and differences related to the orientation of the slots (Table 1). The video sequences for Mk‐CA and Mk‐LO (current study) and Mk‐JO (Kaeser et al. 2011) (see also http://www.unifr.ch/neuro/rouiller/ACCI/videos.htm) highlight that such an extent of additional functional recovery is qualitatively significant, especially for the ability to perform the most difficult retrieval of pellets from the horizontal slots (a gain of 36% for the horizontal slots in Mk‐CA; 46% in Mk‐JO). Enhancement of such gain of functional recovery may be achieved by extending the ANCE re‐implantation sites to non‐primary motor areas, such as PM or SMA, which contribute to the functional recovery (Liu and Rouiller 1999; Dancause et al. 2005, 2006; McNeal et al. 2010; Dancause and Nudo 2011; Hoogewoud et al. 2013; Murata et al. 2015; Plautz et al. 2016, 2023; Medalla et al. 2020; Moreau‐Debord et al. 2021; Calderazzo et al. 2022). However, treating a healthy cortical sector like PM or SMA, which are involved in plastic changes, is most likely different from a treatment aimed at the peri‐lesional sector, in particular regarding the crucial issue of determining an appropriate critical time window for intervention (Plautz et al. 2023). Moreover, a gain of 20% to 40% provided by the ANCE treatment may allow subjects to reach a performance threshold above which the personal motivation to perform intensive rehabilitative training is substantially increased. Additionally, combining the ANCE treatment with neuro‐rehabilitative training (Darling et al. 2024) and/or epidural or subdural electrical stimulation (Plautz et al. 2016; Khanna et al. 2021) may produce synergistic effects. In addition to the gain of manual dexterity performance (as assessed by the score; see Figure 3), after the ANCE treatment the two monkeys Mk‐CA and Mk‐LO exhibited an additional change of behavioral strategy (or motor habit; see Kaeser et al. 2013) reflected by a modification of the sequential pattern to visit the different zones of the modified Brinkman board (see Videos 1 and 2). At this step, it remains unclear whether this change in sequential pattern is due to the biopsy in the prefrontal cortex (Badoud et al. 2017) or to the re‐implantation, or both.

### 4.2 Limitations of the Study

The present report addresses the limitations of our earlier pilot study (Kaeser et al. 2011), which was restricted to only two ANCE monkeys (compared to five control monkeys). The addition of the current study, adding two new ANCE monkeys as well as one new control monkey, confirms the strong contrast between the two groups of monkeys regarding the time course of functional recovery (two plateaus vs. one plateau) and strengthens the conclusion that ANCE significantly enhanced functional recovery, with a reasonable number of subjects (*n* = 10). This represents a substantial sample size considering the current ethical concept of reducing the number of non‐human primates enrolled in experimental studies as much as possible. Nevertheless, a further limitation of the present study is that the four ANCE monkeys fall within a fairly restricted range of small to medium M1 lesion volume (19–30 mm^3^; Figure 4), whereas the range for the control monkeys was larger (13–113 mm^3^). As a consequence, the positive enhancement effect on functional recovery of the ANCE treatment has been demonstrated in the case of small‐ to ‐medium sized lesion (up to 30 mm^3^). Its possible benefit in case of a larger lesion remains, at that step, an open question, and, if it does enhance recovery, to what extent? Another limitation of the present study is that the precise cellular mechanism of action of the ANCE transplant remains largely unknown. Moreover, considering that female macaques recover somewhat better and faster than males (Bottenfield et al. 2021), the sex may moderately affect the present results (Table 1): in the ANCE group, there were 2 males and 2 females, whereas in the control group, there were more males (*n* = 4) than females (*n* = 2). This distribution may have slightly favored the ANCE group but most likely does not account for the considerable recovery disparity between our two groups of monkeys with regard to the occurrence of a second plateau in the ANCE group only. Finally, it has also been shown that monkeys aged 6–9 years old (still considered young) recovered more quickly from M1 lesions than middle‐aged monkeys (14–20 years old: Moore et al. 2012). In our population of monkeys (Table 1), three of them were older, 11–14 years old (Mk‐CA, Mk‐LO, and Mk‐CA), than the others (3.4–5 years old). However, two “old” monkeys were in the treated group, whereas the control group included only one “old” monkey, and such an age factor would then be more detrimental for the treated group than for the control group. In the treated group, the two females are clearly older than the two males. The disadvantage of the advanced age of these females may however, be, at least in part compensated by the more efficient and rapid functional recovery reported in females as compared to males (Bottenfield et al. 2021). In the general context of manual dexterity as a function of age in macaques, a mild impairment of manual dexterity was reported in aged monkeys (15–26 years old) as compared to younger monkeys (9–12 years old; Moore et al. 2010). The three oldest monkeys in the present study (11, 11.5, and 14 years old) correspond to an age range in between the two age cohorts of Moore et al. (2010), suggesting that a putative age effect on these three monkeys is most likely modest.

### 4.3 Mechanisms of ANCE Effects and Comparison With the Literature

The reimplanted cells were labeled with a fluorescent marker in order to tentatively identify them post‐mortem on histological sections (Kaeser et al. 2011; Brunet et al. 2005; Borgognon et al. 2017, 2019). Unfortunately, the extended time gap between the reimplantation and the sacrifice of the monkeys at the end of the experiment (about 6 months up to 1 year; see Figure 3; Table 1) resulted in a loss of the fluorescent labeling in the present study, in contrast to a short time gap that was applied in early experiments in the absence of behavioral follow‐up (Brunet et al. 2005). As reported earlier (Kaeser et al. 2011), we have observed SMI‐32 positive neurons in the M1 lesioned territory in the ANCE monkeys, absent in the lesion area of control monkeys. At that step, it is not known whether the implanted ANCE cells, which survived and migrated, establish new connections, stimulate reconstruction of local neural circuits, or promote cellular and/or synaptic plasticity, as previously reported (Hermann and Chopp 2012; Ueno et al. 2012; Chen et al. 2014; Zhang and Chopp 2015; Zhang et al. 2016; Medalla et al. 2020; Liu et al. 2021; Zhou et al. 2023). Other indirect mechanisms may come into play, such as modulation of inflammation (Moore et al. 2019; Go et al. 2020; Calderazzo et al. 2022; Mccann et al. 2025), enhancement of local angiogenesis (Xiong et al. 2010; Moore et al. 2016; Zhang et al. 2016), effect on myelin and glial cells (Orczykowski et al. 2019; Go et al. 2021), decrease of injury‐related pathologic changes in cortical motor circuits (Medalla et al. 2020), and/or delivery of trophic factors (Moore et al. 2013; Bang et al. 2022), as also discussed previously in earlier ANCE reports from our laboratory on cortical lesion or Parkinson's disease non‐human primate models (Kaeser et al. 2011; Borgognon et al. 2017, 2019).

The present results are consistent with previous cellular therapies conducted in non‐human primate models of motor cortex lesion (Moore et al. 2019, 2013; Medalla et al. 2020; Kim et al. 2025), which similarly produced an improved functional motor recovery, although in the latter studies the treatment strategy was heterologous (human umbilical tissue‐derived cells or exosomes/extracellular vesicles derived from monkeys’ mesenchymal stromal cells, respectively), in contrast to our study based on autologous cell transplantation. An enhanced functional recovery following motor cortex lesion in non‐human primates was also observed after treatment with inosine (Moore et al. 2016).

The vast majority of the monkeys involved in the present study recovered a pattern of grasp movements that was close to the pre‐lesion precision grip sequence, with the noticeable exception of the monkey Mk‐CE, the animal with the largest M1 lesion and limited functional recovery, which exhibited a strategy of substitution consisting of compensatory movements (change in index‐thumb oppositions). This observation is in line with a previous motor cortex lesion study (Murata et al. 2008), reporting greater recovery when the original motor strategy was restored. Restoration of preoperative grasp patterns after M1 lesion, especially in the ANCE‐treated monkeys, is consistent with a comparable observation in monkeys subjected to motor cortex lesion also treated with a cellular (but heterologous) therapy (Moore et al. 2019), in contrast to their control monkeys. Except for Mk‐CE, our control monkeys also largely returned to pre‐lesion grasp patterns, as for the ANCE treated monkeys. The difference between our control monkeys and those of Moore et al. (2019; see also Moore et al. 2012) may be due to larger motor cortex lesions in the latter study. Furthermore, aging (macaques of 14–20 years old) may favor the occurrence of compensatory movements in the functional recovery of manual dexterity after M1 lesion (Moore et al. 2012).

## 5 Conclusion

In conclusion, the present study confirms that ANCE treatment provides a substantial enhancement of functional recovery of manual dexterity after lesion of the hand representation in M1. These findings, considered alongside those of the previous ones (Kaeser et al. 2011), are now supported by a reasonable cohort of non‐human primates to strengthen their validity. This conclusion is based on two pieces of evidences. First, there is a complete dissociation between the two groups of monkeys (four ANCEs versus six controls) in terms of the presence versus absence of a second plateau of functional recovery. The link between a second plateau and a beneficial treatment was also observed for the same M1 lesion model after an anti‐Nogo‐A antibody treatment (Wyss et al. 2013). Second, the temporal unfolding of the ANCE action (about 3 months) was consistent in the four ANCE monkeys, suggesting a reproductive effect across individuals. As recently reviewed (Rouiller 2024), the current ANCE treatment, as well as the anti‐NogoA antibody treatment, modified the density of corticofugal projections from PM and/or M1 in monkeys subjected to motor cortex lesions or Parkinson's disease symptoms. These findings represent a possible substrate for a part of the therapy‐enhanced functional recovery of manual dexterity.

Building on the present behavioral findings in monkeys in conjunction with earlier reports (Brunet et al. 2003, 2005, 2009; Bloch et al. 2014, 2011; Kaeser et al. 2011; Borgognon et al. 2017, 2019), as well as on a large amount of detailed procedural descriptions for the production of cells in GMP conditions, the Cell Production Center of the Lausanne University Hospital (Dr. Brunet and Prof. Bloch) obtained the approval from the Swiss Medical Regulation Office (SwissMedic) to initiate clinical trials to assess the possible benefit of the ANCE treatment in stroke and/or Parkinson patients, as conducted recently in stroke patients with an autologous mesenchymal stem cells approach (Bang et al. 2022).

## Author Contributions

EMR, ES, MK, JFB, and JB designed the present study. CR, MK, and JS trained the monkeys and performed the behavioral sessions. CR, MK, JS, EMR, and ES analyzed the behavioral data. JFB and JB produced the autologous cells for the ANCE transplantation. JB, ES, and EMR performed the surgeries. CR, MK, and ES performed the ICMS sessions and injections of ibotenic acid in M1. EMR and ES prepared the final figures. EMR drafted the manuscript, which was revised and approved by all authors. EMR performed the post‐review revision.

## Ethics Statement

The animal experiments were covered by official veterinary authorizations delivered by Federal (Swiss) and Cantonal (Fribourg) veterinary authorities (see last paragraph of Subsection 2.1 above for detail).

## Conflicts of Interest

The authors declare no conflicts of interest.

## Data Availability

The raw data are accessible on request via the corresponding author.
